# Use of Social Comparisons in Interviews About Young Adults’ Experiences of Chronic Illness

**DOI:** 10.1177/1049732314553122

**Published:** 2015-03

**Authors:** Janet Heaton

**Affiliations:** 1 University of Exeter, Exeter, UK

**Keywords:** adolescents / youth, concept development, illness and disease, chronic, qualitative analysis, theory development, young adults

## Abstract

In this article I examine how young adults used social comparisons in research interviews about their experiences of chronic illness. The interviews were originally conducted not only to provide data for academic analysis but also to generate experiential accounts for publication online as part of an Internet-based health information resource for patients, professionals, and the public wanting to learn about people’s real-life experiences of illness in the United Kingdom. Through secondary analysis of these data, I show how the young adults used various social comparisons to represent themselves and their experiences to the target audience. Two new concepts—analogues and foils—are introduced to describe how the young adults likened themselves to, and contrasted themselves with, different reference groups in their accounts. Through these and related strategies, they created positive renditions of their experiences for the audience, helping to inform and support others in the process.

Originally developed by Leon Festinger (1954), social comparison theory is essentially concerned with the ways in which people compare themselves to others and to themselves at different points in time, and the consequences of such comparisons. The theory has much in common with reference group theory pioneered by Hyman and Singer (Hyman, 1942; Hyman & Singer, 1968) and advanced by sociologists such as Merton (1949/1968). It is not my intention to describe these theories and their myriad applications in detail here; reviews of social comparison theory and its uses are already available (Arigo, Suls, & Smyth, 2014; Buunk & Gibbons, 2007; Goethals, 1986; Suls, Martin, & Wheeler, 2002; Suls & Wheeler, 2000). However, I will briefly describe some of the key concepts that I draw on in this article and the ways in which they have been used in existing health research, to provide the background to the present study and the questions it addressed.

A key concept developed from Festinger’s (1954) work is that of upward comparisons, whereby individuals compare themselves with others who are perceived to be relatively better off. Festinger thought that this tendency was associated with a drive humans have to upwardly evaluate themselves in relation to others’ performance (Buunk & Gibbons, 2007). Subsequently, individuals were also found to make downward comparisons to others thought to be worse off or less fortunate. According to Goethals (1986), this type of comparison is more likely to be made by people who feel threatened, or who have low self-esteem, to enhance their personal sense of well-being. Wills (1981) proposed that the same affect was achieved by making lateral comparisons to others seen to be at a similar level in coping with problems; this has also been referred to as parallel comparisons (Bellizzi, Blank, & Oakes, 2006).

Another set of concepts has been developed that refer to social comparisons made over time. Albert (1977) used the term ‘temporal comparisons’ to describe how people self-evaluate their own performance at different points in time. Finally, the concept of trajectory comparisons has been used to describe how people compare the shifting states of others over time (Dibb & Yardley, 2006).

The above conceptual framework has been used in studies examining which types of social comparisons people with medical conditions use and what they achieve in the process. This work has shown that these individuals often make downward comparisons to others perceived to be worse off (Affleck, Tennen, Urrows, Higgins, & Abeles, 2000; Kay, Davies, Gamsu, & Jarman, 2009; Prout, Hayes, & Gelder, 1999; Rasmussen, O’Connell, Dunning, & Cox, 2007; Schur, Gamsu, & Barley, 1999), and that they make comparisons on a range of dimensions (Buunk, Zurriaga, Gonzalez, Terol, & Lopez Roig, 2006). The effect of making downward and upward comparisons has also been found to vary, depending on how the comparison is construed; a downward comparison might, for instance, be positively invoked to suggest that there are others worse off, or negatively proffered as a sign of how things might get worse (Buunk, Collins, Taylor, VanYperen, & Dakof, 1990; Dibb & Yardley, 2006).

Although providing such important insights, existing research on social comparisons has tended to be dominated by psychological interests and approaches. The aforementioned studies in health, for example, have tended to focus on the individuals making social comparisons and the affective consequences for them. They have added to understanding of whether, in making particular social comparisons, people achieve a more positive view of their situation, or whether it helps them to reduce their anxiety knowing that there are others in a similar position to themselves. In contrast, there is a dearth of research undertaken from a more sociological perspective on the routine and relational use of social comparisons in various social contexts. There is therefore a gap in knowledge about how people use different types of social comparisons in particular social situations, and to what effect for both the person making the comparison and others involved in the interaction.

Likewise, existing research on the topic has typically been carried out in laboratory settings, based on experimental designs and using psychological scales or vignettes to collect data on individual attitudes. Fewer studies have used qualitative methods of analysis—such as conversation analysis, content analysis, thematic and narrative analysis—to examine how and to what ends social comparisons are routinely used in social interaction and in different types of social accounts, with some notable exceptions described below.

Recent studies have begun to focus more on the routine use of social comparisons in particular social contexts using a wider range of research designs and methods. For example, Bellizzi et al. (2006) used content analysis to examine what types of social comparisons were used in 30 autobiographies written by adult cancer survivors, and the consequences for them. Although innovative in using this source of data to investigate what the narratives revealed about the “non-reactive, self-generated” (p. 780) use of social comparisons in these accounts, the researchers still focused on the psychological outlook and adjustment of the individuals who produced the accounts. However, in finding that this group used more parallel comparisons and fewer downward comparisons than previous research had indicated, Bellizzi et al. raised the question of whether this might be indicative of differences in the ways people use social comparisons in a “free narrative” (p. 781) such as the autobiographical account.

In another study, researchers examined how people with motor neuron disease (MND) and Parkinson’s disease spoke about the value of peer support and the impact of seeing others with the same condition as themselves in research interviews about their experiences of living with these conditions (Mazanderani, Locock, & Powell, 2012). Through secondary analysis of these qualitative data, the researchers showed how the participants made complex comparisons with others with the “same” condition as part of the work they did to manage identity tensions and define themselves as “being differently the same” (p. 549).

In a related study, Locock and Brown (2010) examined the attitudes of people with MND and their caregivers about meeting and making visual comparisons with people with the condition at support groups as opposed to contacting others “in the same boat” (p. 1502) via online peer support groups. Their attitudes were shown to influence their strategies for seeking or avoiding particular modes of peer support. Although this study again focused on the attitudes of the people making the comparisons, Locock and Brown noted the limited way in which social comparison theory recognizes the possibility of the satisfaction that people might get in helping others, alluding to the still-to-be-explored relational aspects of the use of social comparisons in this and other social contexts.

Building on these studies, in this article I examine the ways in which young adults used social comparisons to represent themselves and their illness experiences in research interviews about living with a chronic illness. As Radley and Billig (1996) and others (Blaxter, 2004; Bury, 2001; Hydén, 1997) have pointed out, illness accounts are not simply windows on inner attitudes and personal beliefs about health and illness but are social constructs, reflecting the ideology and moral conditions of the time in which the narrator and the audience are situated. Accordingly, “the study of accounting involves examining how people are using beliefs and what they are doing when giving their beliefs in particular situations” (Radley & Billig, p. 224). Furthermore, as Frank (1993, 1995) and others (Cornwell, 1984; Kleinmann, 1988; Riessman, 1990) have shown, illness accounts are shaped by the narrator’s perspective, what he or she is trying to accomplish through the account, and whether he or she thinks it will be acceptable to the audience. By studying how such accounts are constructed, we can gain insights into the meaning of illness for people and into how to respond to their needs (Charon, 2006; Greenhalgh & Hurwitz, 1999).

The research interviews examined in this article were more publicized accounts of illness than traditional research interviews. As I explain in more detail below, the interviews were drawn from an existing series of national studies that were purposely designed to collect first-hand accounts of people’s illness experiences not only for academic analysis but also for publication online as part of an Internet-based health information resource for patients and others interested in learning more about people’s real-life experiences of illness and health care. Thus, the participants in these studies were aware that the interviews were being recorded for this educational purpose and that selected excerpts would be made public on a Web site to illustrate the range of views expressed. These interviews therefore provided an opportunity to explore how social comparisons were routinely and relationally used in this particular social context.

## Methods

In recent years, social scientists have made increasing use of data from previous qualitative research, using these data to address new or emergent questions in secondary studies (Heaton, 2004, 2008). The present analysis of social comparisons was undertaken as part of a wider secondary study I conducted using existing qualitative data on young adults’ experiences of growing up with a chronic illness. Below I describe the data I used in the secondary study and how I generated the present in-depth analysis of the use of social comparisons in this context.

### Source of Data

The source of data for the secondary study was a collection of national qualitative studies carried out by the Oxford Health Experiences Research Group (HERG) since 2000, examining people’s experiences of illness and health care in the United Kingdom. These studies form a series that was designed to capture and share people’s real-life experiences of illness and health care. In each study, a diverse sample of 30 to 40 people with a particular medical condition was interviewed about their experiences of living with and managing their condition; the interviews were video- and/or audio-recorded. The topic guide for the interviews was adapted from study to study but had a common framework, reflecting the generic purpose of the studies; hence, a number of similar topics were explored across the different condition groups covered by the series to date.

Findings from the HERG studies were disseminated via the www.healthtalkonline.org Web site, for other patients and their families, the public, and professionals to learn from the participants’ first-hand accounts. The Web site contains thematic summaries of key topics, supported by video, audio and/or written excerpts from the interviews. The full interview transcripts were also anonymized and archived by the University of Oxford, where they are available by request for use in secondary studies such as this one, subject to a license agreement.

### Secondary Study Aims, Sample, and Data

After scoping the data in the HERG collection and discussing my ideas for secondary research questions with the primary researchers who carried out the interviews I was interested in using, I designed a secondary study on young adults’ experiences of growing up with a chronic illness and, in particular, their sense of mastery of their condition (Heaton, 2014). The secondary study aimed to examine what it meant to young adults with various medical conditions to control and master their chronic illness, whether they claimed to have ever achieved this sense of mastery, and how they accounted for achieving a positive or negative sense of mastery over time. I selected three of the HERG studies for these purposes, on young people’s and young adults’ experiences of diabetes (type 1), epilepsy, and a range of long-term conditions. These studies were carried out in the United Kingdom between 2006 and 2008 and the original results were disseminated via the healthtalkonline.org Web site.

The three study samples were each diverse in terms of age, gender, and length of experience of the individual’s respective condition. After excluding 2 participants who had developed their condition after their teen years, the secondary analysis included 102 interviews with 103 people (one was a joint interview with twin brothers). Of these, 61 were women and 42 were men; 40 had epilepsy, 39 had diabetes, and 24 had other long-term conditions (including juvenile arthritis, cystic fibrosis, asthma, eczema, sickle cell disease, scoliosis, and others). The participants were aged 15 to 29 years at time of interview (average = 20 years) and 0 to 19 years at onset of their condition (average = 10 years); they had lived with their condition for 1 to 25 years (average = 11 years from reported onset).

The interviews for these studies followed a similar protocol. Initially, the participants were invited to speak freely about their experiences of living with their condition. They often began by explaining how they had learned they had their condition before moving on to discuss what it was like living with it through childhood and/or adolescence. The interviewer then asked questions using a semistructured topic guide, which included some standard topics across the three studies. For instance, the participants were usually asked if they had any advice or messages for others who had the same condition, and for professionals who cared for them.

### Secondary Data Analysis

I used a combination of thematic and narrative analysis along the lines described by Flick (2006) in the secondary analysis of young adults’ sense of mastery of their condition. This involved reading each of the interviews and preparing case-by-case summaries of the biographies, noting key themes relevant to the research aims in the process. I then compiled a coding framework to index these themes on a computer, using specialist software to facilitate the analysis of common and divergent themes within and across the condition groups. The results of the thematic analysis were then used to purposively select individual transcripts for more detailed narrative analysis, to compare their illness trajectories and the ways in which the participants accounted for shifts in their sense of mastery over time.

One of the themes identified through this process was social comparison. In the thematic coding framework, all examples of social comparisons were initially indexed using a generic code for “other” relevant themes. After all the interviews were indexed, I retrieved the material under this code and reread it. Eight new subcodes were developed and assigned to these data, one of which was a specific code for social comparisons, which I used 293 times. When I retrieved the material so coded I found it to be very rich, including different types of social comparisons. I carried out a third round of coding, this time rereading the full transcripts and applying four new codes, including two dedicated to the types of social comparisons examined in this article—”analogues” and “foils”—which I define below.

This process resulted in a total of 322 segments being indexed using the codes “analogues” and “foils.” I retrieved this material and summarized it by hand in a grid showing which types of social comparisons were made, by whom, and in relation to which reference groups. The results of this dedicated analysis of social comparisons has been written up separately in the present article because of the amount and depth of the material on the topic, and to allow space to locate the findings in the context of previous research on social comparisons. Further information is available elsewhere on the development, design, and conduct of the overall secondary study (Heaton, 2014).

In the present article I focus on the question of how the young adults used social comparisons in their accounts to represent themselves and their experiences of living with a chronic illness to a virtual audience of their peers, health care professionals, and the public. The excerpts used to illustrate the findings have been selected from as many interviews as possible.

### Ethics

The primary researchers at Oxford obtained ethics approval from a National Health Service (NHS) research ethics committee for the original research and for sharing the anonymized transcripts with other researchers under a license agreement. I also submitted the protocol for the secondary study for ethics review by a university research ethics committee; after it was reviewed, the committee concluded that ethics approval was not required. The primary researchers obtained informed consent in writing from the study participants prior to the interviews. Participants were also given the opportunity to check and edit their transcript and approve the final version; their separate permission was also sought for the publication of given excerpts from their interview on the healthtalkonline.org Web site.

## Findings

I found that two broad types of social comparisons were used in the interviews. In one type, the young adults compared themselves with members of particular reference groups which they characterized as being similar or equivalent. I refer to people compared in this way as “analogues,” meaning “a person or thing seen as comparable to another” (analogue, Oxford dictionaries online, 2014) and “something analogous or similar to something else” (analogue, Merriam-Webster dictionary online, 2014). In the other type the young adults compared themselves to members of reference groups they designated as being different in some respect. I refer to people so contrasted as “foils,” meaning “a person or thing that contrasts with and so emphasizes and enhances the qualities of another” (foil, Oxford dictionaries online, 2014) and “someone or something that serves as a contrast to another” (foil, Merriam-Webster dictionary online, 2014).

Although the use of analogues, by definition, involves lateral comparisons to others perceived to be equivalent, and foils entail the use of upward or downward social comparisons to others regarded as different, these new concepts are introduced in this article to draw attention to the ways in which these juxtapositions were constructed in the accounts and used strategically by the young adults to relate their experiences of chronic illness to others. Below, I show how the young adults elected to compare and contrast themselves to three reference groups— others without a medical condition, others with a medical condition, and themselves at an earlier age—and how they represented themselves and their experiences to the intended audience of the interviews in the process.

### Analogues

#### Comparisons to others without a chronic illness

In the interviews, the young adults talked expansively about their experiences of growing up with a chronic illness and how their condition had affected their life. Although many had experienced difficulties, they often gave positive accounts of how they had nonetheless learned to live with their illness and offered advice or sent messages that were intended to help others learn from their experience. One of the ways in which the young adults constructed positive renditions of their experiences was by comparing themselves to their friends who did not have a chronic illness, invoking them as analogues to claim that they were leading similar lives. This is illustrated by the following example, in which a teenager who had lived with diabetes for 14 years claimed to be living “a normal life” like her friends after overcoming problems with giving herself insulin injections:

Like it took me over a year and a half just to eventually inject. So you’ve got to keep, keep going on, and eventually you—I feel that I’m like my friends now. I live a, I live a normal life, like them, and I can do exactly what they do. And eventually you’ll feel like that if you just, if you just keep on going.

Here, and in some of the other excerpts that follow, she directly addressed the audience, assuring them that they, too, could master it if they persevered.

In a variation of the above, the young adults sometimes made comparisons in a way that slightly qualified their similarity to their nondisabled peers but still claimed to be almost the same as them, or near normal, as in these excerpts:

Interviewer (I): What about things like going out, or drinking alcohol, or things like that? Do they, does epilepsy affect your decisions there or not?

Participant (P): I must say it doesn’t. I probably should be more responsible as far as that goes, but then . . . the reason I don’t think of it as much as I probably should is that it’s never posed a problem. I enjoy a night out; I’ll enjoy a glass of red wine or three. Um, it’s, it’s never posed a problem. So I feel perfectly comfortable with it, as I say, this is what I say, I live like anybody else, nearly.

I’ve achieved everything else in my life that I wanted to. I’ve felt that I have been normal to an extent. . . . It’s been great that I’ve been able to do so many things um, and feel so equal to all of my friends.

By invoking their friends as analogues of themselves, the two young women quoted above were able to normalize their respective experiences of epilepsy and cystic fibrosis, suggesting to the audience that it was still possible to do the same things as people who did not have these conditions.

Sometimes the young adults used analogues in combination with other types of social comparisons to make complex claims. Two teenage brothers, who had both had diabetes for 9 years, followed up their general claim of parity with people who did not have diabetes with a downward comparison to a specific individual from the latter reference group, suggesting that the brothers were able to do even more than some such people:

I: At any point in your lives, have you sort of kind of had doubts of uh, or would I be able to do this or not able to do that?

P1: No, not really.

P2: Not really, no. We’ve always been encouraged and we’ve been fairly optimistic about everything we do. So, uh no.

P1: It was part of the reason we went on the trek. Uh, just to prove to ourselves that, you know, as diabetics we can do everything that a person who isn’t diabetic can do.

P2: And better.

P1: Yeah, and better. There was one girl; she wasn’t diabetic but she hadn’t done any training at all.

Further examples of different types of social comparisons used in combination to make complex claims are described and discussed later in the article.

#### Comparisons to others with the same or another medical condition

Whereas the young adults often likened themselves to their friends and peers who did not have a chronic illness, they seldom referred to others with the same or another medical condition as analogues of themselves. This might reflect the view, expressed by a number of the young adults, that they did not like being defined in terms of their condition.

When the young adults did occasionally compare themselves to others with the same or a similar condition, it was usually to suggest that they were not alone and that many people lived with one condition or another, making it mundane. This is illustrated by the following excerpt, from an interview with a young woman who had lived with diabetes for 13 years: “It’s just part of me and it’s part of me that I would choose not to have but, you know, other people are dyslexic or whatever, and ‘so what?’ kind of thing.” In the following excerpt, a teenager who had had asthma for 13 years used a similar strategy. He directly addressed members of the audience with the same condition, assuring them that they were not alone:

Just, just remember that there’s kids out there, loads of kids out there who have the same like, um, who take the same medicines as you and have the same disability as you, so you’re not the only one really.

A teenager who had had diabetes for 4 years also likened his condition to other chronic illnesses that affect young people:

And it takes a long period of time, like several months, to gain the confidence in the fact that it’s only a condition like any other, like any other condition, such as like asthma or even like an al—allergy or something like that. It’s, it’s something small that shouldn’t affect your life, um, and doesn’t affect your life in any significant way.

By so constructing their accounts the young adults appear to have been minimizing the uniqueness and significance of their condition, suggesting that it was one of a number of conditions that many people had and could adapt to over time.

#### Comparisons to self

As well as comparing themselves to others with and without a chronic illness, some of the young adults also made self-comparisons, referring to themselves at different points in their illness biography. In the following example, a young man diagnosed with rheumatoid arthritis early in life portrayed his own experience to be relatively continuous, in contrast to what he imagined it must be like for people diagnosed later in their youth:

Because I have only had it since I was two, I mean, I don’t know any different. So in effect I have never been in the playground playing football. So you know, I have never experienced being normal, although I am normal in lots of respects, you know. I am not, you know, I can’t run around and play football. So in that respect I have never experienced that. So I would say that what you don’t have you don’t miss. . . . There are people out there who have been nondisabled up until they are twelve, thirteen, and then they have become disabled, so I could imagine that would be quite hard. Because you have had all those years of normality and you have grown. I mean, I have never really grown because I got it when I was two. But there are people out there I know that have become disabled at twelve, thirteen, fourteen, so they have had that chance to grow. They have had that chance to interact with people and then they have become disabled. I could imagine that that would be very hard. But I don’t know what it is like because I don’t know.

Using a temporal self-comparison, he indicated that he had no experience of living without his disability and so did not know any different way of life. His present and younger selves were represented as constant in this respect. At the same time, he used others whom he supposed had a more disrupted biography as a foil to suggest that it must have been harder for them to adjust to their disability after they had experienced living a so-called “normal” life.

One young adult who had lived with diabetes for 16 years claimed he was glad that he had acquired the condition relatively early in his life, giving him time to get used to it from a younger age rather than, say, in his teens:

I mean, at the end of the day I’m glad I got it when I was seven instead, and not when I was eighteen, if I’m honest. I’d much rather, at least I knew I had it and I was used to it by then instead of getting it when you’re eighteen and when, you know, you’re trying to have a laugh. I think that would be worse.

In these ways, the young adults who had lived with their condition from a very young age suggested that their experience of chronic illness was relatively continuous and easier to adjust to, and not as disruptive or life changing as they imagined it was for others who developed their condition later in their youth.

### Foils

#### Contrasts with others without a chronic illness

Although some of the young adults portrayed themselves as analogues of their nondisabled friends, emphasizing similarities in their ways of life, others instead contrasted their experiences with the same reference group, using them as a foil to highlight differences in their lives. The following excerpt, from an interview with a young woman who had sickle cell anemia, illustrates this:

And sometimes you just want to, you know, you just want to be like all your friends. But then you have to remember, you know, none of your friends have sickle cell [anemia]; you’re the one that has sickle cell. So they can do things like freeze to death [laugh] and stay up all night and get no sleep, and you know, all they have is a hangover, or they might get a little bit of a cold, but the effect that your body will have is that it will just go into a crisis.

Here, the young woman acknowledged the tension that those with sickle cell anemia experienced in wanting to be like their friends, but having to remember that they were different with respect to how their bodies reacted to living the same kind of lifestyle.

Some of the young adults also talked about the difficulties of trying or being expected to live a “normal” life with their condition:

I’m constantly being told that I need to be enjoying myself. I need to be doing things like normal under-thirty-year-olds should be doing . . . even though that person, people understand that I can’t do that. I should be [sigh] having a laugh. . . . You can’t do it. So cut yourself some slack. Do what you want to do and what you can do and what you know your limitations are. Don’t live to other people’s expectations because you will make yourself ill. If not physically, [then] mentally, because it’s exhausting living to other people’s expectations when you’re ill. At any level, at any time when you’re not ill, it’s exhausting. But when you are ill you can’t do it. It completely and utterly annihilates you. You can’t, you just can’t do it. . . . If you can’t do it, don’t do it. Do what’s important to you. I don’t know. It’s hard though—very.

In this excerpt, a young woman who had lived with chronic pain for 14 years actively distinguished herself from her healthy peers, using them as a foil to emphasize how she found it difficult living up to other people’s expectations about how she should be living her life at her age. For her, this had been unrealistic and damaging. By incorporating the use of upward social comparisons with negative connotations, she portrayed herself, and others in a similar situation, as having a less easy and more limited life in some ways relative to their healthier counterparts. At the same time, she implied it was helpful knowing and accepting her limitations and not trying to live up to other people’s expectations.

Although the young adults who contrasted their experiences with those of their friends and peers usually portrayed themselves as being more limited in some respects, this was not always the case. Occasionally, the young adults made downward social comparisons to nondisabled others, whereby they represented themselves as being somewhat different from these individuals, but in a positive way, as in a teenager’s message to others with the same condition:

Yeah, I’d just like to, you know, encourage you and say that just because you have sickle cell doesn’t mean that you’re not normal. Do you understand? It’s like we are, we are different, yeah, but privileged in a way to say that we have sickle cell and have an understanding and a different side to see things from. So that’s why I encourage you and keep going, you know, doing energetic things.

In these various ways, some of the young adults distinguished themselves from their nondisabled peers and suggested that, despite the desire and expectation to be normal, they had learned to accept their differences and encouraged others to do the same.

#### Contrasts with others with the same or another medical condition

When the young adults referred to others with the same or another medical condition, it was usually to contrast themselves with and differentiate themselves from this reference group, rather than depicting themselves as analogues of them. This reference group was commonly invoked as a foil in two ways. The first was in mostly downward and positive comparisons, whereby the young adults claimed to be better off or “lucky” compared to others with the same or another medical condition, as the following examples show:

But, but then my experience of it [diabetes] is, as I said, has been better than a lot of people’s, so I don’t want to, I don’t want to sit here and, and seem like I’m coming across like really arrogant and, um, you know, condescending to other people. Because I know people have it a lot harder than I, than I do. And I know people’s diabetes is a lot harder to control than mine is. And I am lucky how I can, I can get away with things.

I: How serious a condition do you think personally that epilepsy is?

P: Depends on how controllable it is. Em, as I said earlier on, I’m very lucky. I’m very fortunate in fact that everything has smoothed out. Em, if you’re photosensitive I, I really feel sorry for you.

I mean, another thing about my motivating myself is there’s always someone worse off than me, so I’m not going to complain about it [diabetes]. I’ve got it quite, really, really good compared to a lot of other people, so it’s only a couple of injections and needles a day. There is nothing compared to the hundreds of tablets cancer patients—and the radiotherapy and everything that they have to suffer, and the starving children. So I’m, I’m perfectly well compared to a lot of other people, so don’t complain and get on with it.

In the above excerpts, the young adults (2 men and 1 woman) differentiated themselves from others with the same chronic illness or another condition on a range of dimensions—control, triggers, burden of the regime, and suffering. They acknowledged that experiences vary and that some people might have a more difficult time than they claim to have had.

There were just a few exceptions, when the young adults made upward comparisons to others with the same condition, describing how they had felt different and “unlucky” compared to them. In the following excerpts, a teenage girl with diabetes and a young man with epilepsy portrayed themselves as having relatively unusual or exceptional experiences of controlling their respective conditions, the first because she had difficulties injecting herself when younger and the second because the medications he had tried had not worked for him:

I: Before you started injecting yourself did you feel different?

P: Yeah. Um, especially—yeah, especially amongst other diabetics as well. Like I knew that—because we had friends that were diabetic and one of them’s been injecting since six and—and I knew that. I injected when I was five once but then that was it, and I knew that I, I was quite different to the other ones, but—so it was quite challenging back then. Well, I got quite upset about it, so.

I: So you felt different in relation to other young people with diabetes?

P: Yeah, more the other young people with diabetes more.

I: Rather than your peer group?

P: Yeah, because I knew that they didn’t really have it, whereas other diabetics have had to deal with it, and I thought why couldn’t I, so, so.

To be honest with you, most, most people can get their em, medication to, to work brilliantly and it [epilepsy], it either grows out or they [seizures] completely controlled. I’ve been slightly unlucky. . . . The guy told me if you’re on the wrong medication it can make the epilepsy worse sometimes, em, so it could’ve made it worse. I mean, I believe it did and . . . but I, I definitely got the impression when . . . I had the grand mal seizures that a lot of people have been in my situation and a lot of people live with it brilliantly, especially in my school.

By reporting their negative experiences in this way, these young adults described the difficulties they encountered without giving the impression that they were necessarily typical.

The second common way in which the young adults differentiated themselves from others with a chronic illness was to contrast the ways in which they managed their condition relative to how others managed theirs. Members of this reference group were again invoked as foils in downward comparisons, this time to support counterfactual claims that they were not letting the condition stop them from living their life, and that they were managing their condition better than others were:

I’ve always been pretty confident in that I don’t let it stop me doing things. I hear a lot of diabetics who, they, you know, make sure their life’s very rigid and they don’t go off and do lots of active stuff, or they won’t put themselves in situations where they may miss meals or they may, yeah, go out and drink or party or things. But I have always been adamant that I’m not going to let it muck up my life.

I’ve got friends who just don’t take their Creon at all. They just don’t even bother taking their tablets just because, I don’t know, whether it’s like rebellion or you know, or if they forget, but I’ve never had a problem. It’s always just been habit so, once you get into a routine, just, it’s quite easy just to stick to.

Here, two young women who had diabetes and cystic fibrosis justified their preferred way of managing their respective condition by juxtaposing their approach with those of others, which they did not condone.

#### Contrasts with self

Earlier we saw that the young adults who had been born with their condition, or who had lived with it from a very young age, imagined that it was harder for those who developed it later in their youth to adjust to. However, when those who had developed a chronic illness later in their youth made temporal comparisons to their pre-onset-of-condition selves, they tended to portray themselves as having eventually benefited from their experience, as these excerpts illustrate:

Yeah, and I just think everything in my life did change, and so I think it’s impossible not to change kind of as the result of that, um, but I really think all the changes that happened actually happened for the better. I know that sounds like a really weird thing, you know, that getting epilepsy, well developing epilepsy, you don’t get it, it just happened. Um, you know everybody would see that as a really negative awful thing, and I think I did a little way, well mainly because everything was kind of taken away from me. I resented it for a bit, but actually everything that was taken away um, was replaced with even better things.

[Diabetes has] helped me in lots of ways. Like I said, I quit smoking, I eat healthier diet, I’m doing more exercise than I would have done if I didn’t have the diabetes. . . . I can honestly say that in the time that I’ve had the diabetes I’ve, there’s been less than twenty times in sort of eight years, nine years where I’ve wished that I haven’t got the condition.

In these cases, a young woman and a young man who developed epilepsy and diabetes at the age of 18 and 15, respectively, described the positive ways in which their lives had changed since having their condition.

To a lesser extent, some young adults made temporal contrasts to their former, pre-onset-of-condition selves and lifestyles, with more negative connotations. This is illustrated by the following excerpt from an interview with a young man who developed epilepsy at the age of 15:

I don’t think I’m the same person that I would’ve been if I hadn’t had it. Certainly that. It certainly would have affected my main developmental years. I mean, if you think about it, fifteen to twenty-one, I mean that’s when you become who you are really, I suppose. So I suppose it’s made me, although no one will tell me that, tell you that I am a lot more cautious than I might have been, which means that I really would’ve been wild if I had actually um, just got on with it.

In these ways, the young adults who had developed their condition later in their youth used their pre-condition selves as a foil to highlight the positive or, less often, negative ways in which their life had changed since the onset of their illness.

### Multiple and Compound Comparisons

So far I have separately examined the use of analogues and foils in the research interviews. This is not meant to imply that the young adults used either type of social comparison exclusively. On the contrary, multiple examples of analogues and foils were found in single interviews. The balance varied from account to account, and together contributed to the overall representation of an experience as being positive, mixed, or negative, for the audience to learn from.

In addition, as we have seen, different types of social comparisons were sometimes used close together in sections of an interview to make complex claims. This is illustrated by the following excerpt, in which a young woman who had epilepsy discussed how people with the condition could feel isolated:

I think isolation can be a big one. Um, because having a condition does make you a little bit different. It does, um, and it, it can make you feel different. And particularly when you’re a teenager, you want to be like your friends and you want to look like your friends and do the same thing as your friends are doing. You don’t want to be different. . . . Um, there’s also the issue that um, a lot of people have epilepsy, a lot of people in the UK have epilepsy, but you don’t know by looking at them. So you can, it can feel very, very, lonely, you know, that nobody else understands, nobody else knows, knows what it’s like to have this condition. Um, the chances are that you probably do know somebody with a health condition. You just don’t know by looking at them that they, that they do have one. But, yes, it can feel quite lonely.

Here she constructed her claim that people with epilepsy can feel isolated and lonely by first of all highlighting how they, and especially teenagers, can perceive themselves to be different from their friends because of their condition. This was closely followed by the observation that people with epilepsy also do not necessarily know of others with the same condition because of the hidden nature of the condition. Through these different types of social comparisons, she captured and conveyed the distance from both their friends and from others who have epilepsy or another hidden condition that people with epilepsy can experience.

## Discussion

In this article I have attempted to develop a sociological analysis of the ways in which young adults used social comparisons in research interviews about their experiences of living with a chronic illness. In the analysis I focused on the routine and relational aspects of the use of social comparisons in this context. This has provided new insights into the ways in which the young adults strategically used social comparisons to represent themselves and their experiences to the intended audience of these accounts, which included their peers and health care professionals, as well as primary and secondary researchers.

Through a detailed analysis of the interviews I found that the young adults regularly used two types of social comparisons, which I called analogues and foils, to compare and contrast themselves to others, and to their younger selves. Although these types of comparisons entailed lateral, upward, or downward comparisons that are familiar in the literature, the new concepts were introduced to reflect the new perspective adopted here. This has involved moving away from a predominantly psychological interest in what the direction of the comparison might suggest about the disposition of the person making the comparison, toward a sociological interest in the strategic ways in which these comparisons were used to represent the identity and experience of the person making the comparison to the intended audience of the account.

In contrast to previous studies mentioned earlier, suggesting that people with medical conditions tend to make downward comparisons, in this analysis I found that the young adults used a mix of analogues and foils in their individual interviews (including lateral, downward, and upward comparisons), and that they sometimes used them close together in compound forms to make complex claims. I have suggested that these devices were used to represent themselves and their experiences in particular ways for the intended audience of the accounts, often creating positive renditions of their experiences for others to learn from. This finding is consistent with previous studies in which people reportedly described their experience of chronic illness in positive terms, as well as valuing and identifying a need for more positive and realistic role models of disabled people’s lives (Adams, Pill, & Jones, 1997; Kay et al., 2009; Rasmussen et al., 2007; Schneider & Conrad, 1983; Schur et al., 1999).

In the analysis, I have also suggested that the ways in which the young adults used social comparisons were related to strategies that have been documented in the wider literature on people’s experiences of illness. For example, by portraying themselves as analogues of their nondisabled friends, the young adults were able to downplay their purported difference from others, even when they simultaneously reported having symptoms and difficulties connected with their illness. This finding adds to previous research examining how people normalize and minimize their illness (Atkin & Ahmad, 2000, 2001; Kelleher, 1988; Prout et al., 1999; Schneider & Conrad, 1983; Schur et al., 1999) by showing how invoking analogues plays a part in this process. Indeed, it is worth noting that some of the excerpts published to illustrate the aforementioned work on normalization contain embedded social comparisons akin to those identified in the present analysis, although they are not mentioned (see Kelleher, pp. 39, 50, and 63; and Schneider & Conrad, pp. 74 and 84).

Similarly, the ways in which the young adults used analogues and foils to portray themselves as similar to or different from nominated disabled and nondisabled reference groups can be viewed as part of the work people do to claim or resist alternative candidate identities such as being “normal” or, for example, being “a diabetic” or being “disabled” or “different.” Again, previous research, such as the study by Adams et al. (1997) on the “accepters” and “deniers/distancers” of asthma, has documented this kind of identity work and alluded to social comparisons without looking explicitly at the use of juxtapositions and counterfactuals in these strategies. The same can be said of the role of temporal comparisons in studies of biographical disruption (or flow or reconstruction) following work by Bury (1982), and of people’s sense of loss or (dis)continuity of self after the onset of illness following work by Charmaz (1983) and Riessman (1990).

By examining the ways in which social comparisons were used to account for illness experiences in the social context in which the narratives were produced, I have attempted to develop an understanding of the relational aspects of the use of these devices. In this way, I have tried to add to understanding of the ways in which social comparisons were used from the point of view of both the young adults making the comparisons and the audience to whom the accounts were directed. Although not immediately present, the participants were evidently mindful of the audience when giving their account, sometimes addressing the audience directly. Such remarks were usually directed to other young people and young adults who had a chronic illness, as in several of the excerpts presented above. This was often done spontaneously and not only when they were asked if they had any advice or messages for other young people and young adults. At other times, the young adults addressed health care professionals or parents, sending them messages about how they could best support other young people and young adults in a similar situation.

More generally, those in the audience with the same condition were also tacitly characterized in the interviews as being relatively naïve, in the process of learning about aspects of their condition and how people live with it, from the participant and others who have been in a similar position. The research interviews were therefore founded on a social comparison of a sharing of experience between differentially experienced people who had a chronic illness, and in a way were intended to inform and help the learning audience. In this way, the accounts are a prime example of what Charon (2006) called “narrative medicine,” whereby personal accounts of illness are used to help close the gap in understanding between people with varying experiences of illness, as well as between patients and their families, physicians, other health care professionals, and the general public.

A limitation of this study is that being a secondary analysis it was not possible to ask the participants themselves to reflect on how and why they made particular social comparisons. Although I have offered my own interpretation of their claims, it is possible that their social comparisons meant something else to them. Likewise, no attempt was made to directly ascertain how the various groups accessing the interviews interpreted the social comparisons that were made in this context. How people at different points in their illness trajectory, and with a range of illness experiences, interpret the comparisons, and whether they find them positive and helpful and, if so, why, are important questions. These and related questions would need to be addressed through additional primary research, perhaps using the present findings and other recent work on the routine use of social comparisons as the starting point for such an inquiry.

In addition, although the research interviews used in this study were a very rich source of data for analyzing the routine and relational use of social comparisons, it is possible that the ways in which young adults used analogues and foils in this particular narrative context differ from how they might have used them in other narrative and social contexts, such as research interviews that were not to be shared with others, or in online forums or face-to-face support groups with other people with the same condition, or in consultations with clinicians. Although I have shown that analogues and foils were generally used by the young adults to create positive renditions of their experiences for others to learn from in the particular interviews examined in this article, the same devices could be used by them to create more ambivalent or negative renditions in other contexts.

There is a need for future theoretical and empirical research to consider the routine and relational aspects of the use of social comparisons in different contexts. More attention needs to be given to how analogues and foils are employed by people with medical conditions as part of wider strategies for normalizing illness and managing identity in accounts, as well as conveying a sense of how well a person is coping with and has adapted to his or her condition, in particular social contexts. Such work might usefully examine how people invoke different reference groups as analogues and/or foils, in which narrative contexts, for which audience, and to what effect for both the person making the comparisons and the audience for the account.

For example, if young adults, who might see the onset of chronic illness in their youth as a potential threat to retaining membership of their nondisabled peer reference group, employ analogues as a way of normalizing illness and foils as a way of differentiating themselves from others with their condition, how do older people who develop an illness later in life account for their experience using social comparisons? If, as has been suggested, some people see the onset of illness later in life as a normal part of aging (Pound, Gompertz, & Shah, 1998), perhaps older people compare and contrast themselves to their able and disabled peers in different ways. Furthermore, what do these accounts and the use of social comparisons reveal about social attitudes toward and public understandings of aging, illness, and inequalities across the life course?

By better understanding this, future research could help to inform the design and dissemination of health information incorporating social comparisons. Such information could be used to close the gap in patients’, the public’s, and health care professionals’ understanding of the lived experience of illness, and to help people live a positive life with their illness. As this analysis has shown, how young adults compare and contrast their experiences of chronic illness to various others, and to their younger selves, provides more than an indication of how the person making a social comparison perceives his or her condition and whether he or she has adjusted to it. It also provides a way of helping to inform and support others who might be going through a similar experience by providing positive renditions of even the most difficult experiences, potentially allaying their common fears and concerns about feeling different and disadvantaged by their condition.
